# A novel metacyte metafer classifier for platelet morphology using long COVID as a model

**DOI:** 10.1007/s11239-025-03144-9

**Published:** 2025-07-11

**Authors:** Chantelle Venter, Jan H. Pretorius, Douglas B. Kell, Etheresia Pretorius

**Affiliations:** 1 Department of Physiological Sciences, Faculty of Science, Stellenbosch University, Private Bag X1, Matieland, Stellenbosch, 7602 South Africa; 2 Department of Economics, Faculty of Economic and Management Sciences, Stellenbosch University, Private Bag X1, Matieland, Stellenbosch, 7602 South Africa; 3 Department of Biochemistry and Systems Biology, Institute of Systems, Molecular and Integrative Biology, Faculty of Health and Life Sciences, University of Liverpool, Liverpool, L69 7ZB UK; 4 The Novo Nordisk Foundation Centre for Biosustainability, Technical University of Denmark, Kemitorvet 200, Kgs Lyngby, 2800 Denmark

**Keywords:** Microscopy, Fluorescence, Platelet activation, Laboratory automation (MetaSystems metafer system)

## Abstract

Platelets play a critical role in coagulation and are implicated in pathological clotting. This study analyzes platelet activation and morphology using the cellular fraction remaining after whole blood centrifugation. We briefly examine whether the addition of platelet inhibitors, such as Indomethacin and Prostaglandin E1 (PGE-1), affects platelet activation status during sample preparation. Additionally, we introduce an automated analysis method using the MetaCyte Metafer classifier to enhance objectivity and precision in evaluating platelet fragility and activation. Our disease model includes platelets from participants with Long COVID compared to healthy controls. The automated analysis provided consistent, unbiased data, and detailed metrics such as area, count, and roundness, indicating a significant improvement in platelet analysis. We found that platelet inhibitors did not influence platelet activation during sample processing, suggesting that platelets in Long COVID patients are substantially activated in circulation. Automated analysis using the MetaCyte Metafer classifier offers a reliable approach to identifying subtle morphological changes in platelets, including activation size ranges, which can provide valuable insights into the progression of platelet pathology in Long COVID and other thrombo-inflammatory conditions. Future work should focus on refining algorithms and incorporating artificial intelligence (AI) to enhance automated data interpretation and clinical relevance.

**Supplementary Information:**

The online version contains supplementary material available at 10.1007/s11239-025-03144-9.

## Introduction

Platelets play a crucial role in coagulation, responding to vascular injury by adhering to damaged vessel walls and forming a plug to prevent further bleeding. However, under certain pathological conditions, platelets can become hyperactivated, leading to inappropriate activation and contributing to inflammatory diseases. In conjunction with fibrinogen polymerizing into fibrin, activated platelets can drive the formation of pathological thrombi. Long COVID, also known as post-acute sequelae of SARS-CoV-2 infection (PASC), refers to a range of persistent symptoms and health complications that continue or develop after the acute phase of COVID-19, lasting for weeks or months. Common symptoms include fatigue, cognitive dysfunction, shortness of breath, cardiovascular and clotting abnormalities, and a variety of neurological and inflammatory manifestations. Endothelial dysfunction and hyperactivated platelets are now recognised as key pathologies in Long COVID [1–3]. Platelet activation markers have also been noted in proteomics analysis of samples from Long COVID patients [4, 5].

Several laboratory techniques exist to study platelet characteristics, including numbers, size, and function (see Table 1). Our previous work in acute COVID-19 and Long COVID involved analyzing platelet structures in the cellular fraction of blood, prepared by centrifugation, where platelet poor plasma (PPP) is separated from the cellular fraction [5–7]. In these studies, we used antibodies such as PAC-1 (marking activated platelets by binding to the neoepitope of active GPIIb/IIIa conformation on the platelet surface) and CD62P-PE (marking p-selectin on the platelet membranes). PAC-1 is commonly used to identify activated platelets by binding specifically to the neoepitope of the active GPIIb/IIIa complex on the platelet surface, while CD62P-PE is a marker of platelet degranulation and activation, expressed on the membrane following activation. We showed that platelets in Long COVID are significantly activated, compared to platelets from healthy participants. We have shown that platelets from healthy participants are quiescent, with minimal activation. Recently, Buonsenso and co-workers also examined platelet activation and secretion markers in children, through flow cytometry, utilizing monoclonal antibodies targeting P-selectin, CD63, and PAC-1. Both quiescent platelets and those stimulated with 10 µM adenosine diphosphate (ADP) and 25 µM thrombin receptor activating peptide (TRAP) were analyzed [8]. The mean fluorescence intensity was quantified and represented as a fold increase compared to paired control samples. The findings were reported as medians and ranges [8]. They concluded that, given the increasing evidence of persistent thrombo-inflammation in adults with Long COVID, that activated platelets, also seen in adults, are more prevalent in children with Long COVID compared to controls. These results potentially also indicate underlying thrombo-inflammation and damaged endothelial layers in children.

**Table 1:** Platelet analysis in general pathology laboratories and methods available for research purposes

General pathology tests		
Test	Description of test used	References
Complete Blood Count (CBC)	This test provides information about the number and size of platelets in the blood, which can indicate abnormalities in platelet function.	[18–20]
Bleeding Time Test	This test measures the time it takes for a small cut to stop bleeding. It evaluates the ability of platelets to form a plug and stop bleeding.	[21–23]
Platelet Aggregation Test	This test measures the ability of platelets to stick together in response to a chemical stimulus. It can evaluate the response to antiplatelet drugs, such as aspirin, and identify platelet dysfunction.	[24–26]
PFA-100 and 200 Tests	This test evaluates the ability of platelets to form a clot in a small tube. It can detect platelet dysfunction and is often used before surgery to evaluate bleeding risk.	[7, 27–30]
VerifyNow Test	This is a point-of-care test that evaluates platelet function by measuring the degree of platelet inhibition by antiplatelet drugs, such as clopidogrel or aspirin.	[31–33]
Thromboelastography (TEG) and rotational thromboelastometry (ROTEM)	This test measures the ability of the blood to form a clot and evaluates the contribution of platelets and other factors to clot formation.	[7, 34, 35]
**Research methods**		
Flow Cytometry	This is a powerful technique that can be used to analyse the properties of individual platelets, such as their size, shape, and surface proteins. It can also be used to evaluate platelet activation, aggregation, and adhesion.	[36–40]
Platelet Secretion Assays	These assays evaluate the release of bioactive molecules, such as adenosine triphosphate (ATP) and serotonin, from platelets in response to different stimuli. They can be used to evaluate platelet function and activation.	[41–43]
Microscopy Techniques	Various microscopy techniques, such as electron microscopy and fluorescence microscopy, can be used to visualize platelets.	[6, 44–50]
Proteomics and Genomics	These approaches use mass spectrometry and gene expression analysis to identify the proteins and genes involved in platelet function and activation.	[51–53]
Platelet Transcriptomics	This approach analyses the RNA molecules expressed in platelets to identify genes and pathways involved in platelet function and activation	[54–56]

Here we closely look at laboratory methods used to prepare samples to study platelets, and also investigate the use an automated method for unbiased morphological analysis using microscopy. Activation of platelets due to processing, is an important and fundamental research question when studying platelets. Platelets from Long COVID patients are fragile and the question arises if centrifugation indeed activates them more easily. Addition of platelet inhibitors such as Indomethacin and Prostaglandin E1 (PGE-1), are often used to maintain platelets in a quiescent state and are added shortly to whole blood, after blood is draw. PGE-1’s primary function is to inhibit platelet activation and aggregation, while Indomethacin inhibits cyclooxygenase (COX) enzymes, which reduces the synthesis of thromboxane A2, a potent inducer of platelet aggregation.

Previously, we developed a platelet grading system to assess level of platelet activation [7]. The initial grading system was developed by studying hundreds of micrographs of platelets from healthy participants and Long COVID patients. A number of platelet micrographs were taken systematically (by experienced microscope analysts), over a microscope slide [7]. Sample grading of new anonymized samples then involved taking micrographs over a prepared slide, followed by grading these micrographs according to the developed grading system. However, such a manual grading system can introduce bias to the analysis. Thus, to further improve platelet analysis, here, we introduce an automated platelet analysis method using the MetaCyte Metafer classifier. Metafer is intended for use in in vitro diagnostic procedures by clinical and non-clinical laboratories in accordance with their established procedures [12]. This new automated platelet analysis method provides an objective and unbiased approach to examining platelet activation and morphology. Furthermore, the automated MetaCyte Metafer classifier method allows for precise measurement of platelet characteristics, advancing our understanding of platelet dynamics in Long COVID and other thrombo-inflammatory conditions. This approach enables the study of platelets within a complex cellular milieu, offering novel insights into platelet activation dynamics that may not be captured in platelet-rich plasma preparations.

## Materials and methods

### Blood collection

Ethical clearance for the study was obtained from the Health Research Ethics Committee (HREC) of Stellenbosch University (South Africa) (references N19/03/043, project ID 9521). Four 2.5 mL citrated blood tubes were drawn by a qualified phlebotomist from consenting participants (the first tube that was drawn was discarded). Blood was drawn from healthy controls (*n* = 10, mean age: 29 [± 4]) and Long COVID patients (*n* = 15, mean age: 42 [± 17]) (see Table 2).

**Table 2:** Sample demographics

**Mean age [and standard deviation (SD)] of controls and Long COVID patients**	
Controls (*n* = 10)	29 [4]
Long COVID (*n* = 15)	42 [17]
**Gender of controls and Long COVID patients**	
Controls (*n* = 10)	8 males; 2 females
Long COVID (*n* = 15)	3 males; 12 females
**Vaccination status of controls and Long COVID patients before blood collection**	
Percentage of controls vaccinated (*n* = 10)	90%
Percentage of Long COVID patients vaccinated (*n* = 15)	87%
Symptoms of Long COVID patients (*n* = 15)	
**Symptom**	**% in Long COVID patients**
Brain fog/ concentration issues	53%
Constant Fatigue	67%
Depression/Anxiety	40%
Post exertion malaise after exercise (PEM)	40%
Shortness of breath	27%
Recurring Chest Pain	20%
Heart palpitations	33%
Joint and muscle pain	60%
Sleep disturbance	40%
Digestive problems	20%
Tinnitus	33%
**Co-morbidities of Long COVID patients (*n*** = 15)	
**Co-morbidity**	**% in Long COVID patients**
Periodontitis or gingivitis	7%
Gut dysbiosis	13%
High cholesterol	13%
High blood pressure	7%
Cardiovascular disease	7%
Rheumatoid arthritis	7%

### Sample Preparation and analysis

To confirm that centrifugation alone does not inadvertently activate platelets, blood samples were drawn in citrate blood tubes from one Long COVID individual. Whole blood was centrifuged at 3000 x g for 15 min at room temperature, with and without Indomethacin (10µM final concentration; Enzo, ALX-270-086-G025) and Prostaglandin E1 (PGE-1, 1µM final concentration; Abcam, AB141716) [9–11, 13]. Platelets remaining in the cellular fraction after centrifugation, was the target for the analysis; 20µL of the haematocrit sample was incubated with 4µL CD62P (PE-conjugated) (IM1759U, Beckman Coulter, Brea, CA, USA) and 4µL PAC-1 (FITC-conjugated) (340507, BD Biosciences, San Jose, CA, USA) for 30 min, at room temperature (protected from light). After the incubation, 10µL of the cellular fraction after centrifugation, was placed on a microscope slide and covered with a coverslip. As part of the optimization of the two platelet markers, and as part of the setup of the fluorescence microscope, unstained and single stains were prepared (data not shown).

### Manual fluorescence microscopy analysis

Samples were placed on the microscope slides and viewed immediately after the 30 min antibody incubation using a Zeiss Axio Observer 7 fluorescent microscope (Axiocam 705 camera) with a Plan-Apochromat 63x/1.4 Oil DIC M27 objective (Carl Zeiss Microscopy, Munich, Germany), with the excitation wavelength for PAC-1 set at 450 to 488 nm and the emission at 499 to 529 nm (demarcated as the green fluorescence), and for the CD62P-PE marker excitation was set at 540 nm to 570 nm and the emission at 577 nm to 607 nm (demarcated as the purple fluorescence). Platelet spreading and clumping were quantified by using the grading system, previously developed by our team [7]. Six fields of view were selected as representative images of the sample after thoroughly examining the entire slide manually.

### Novel automated MetaCyte metafer classifier method for platelets

Samples were prepared the same as for the manual method, but after the incubation with the CD62P-PE and PAC-1 markers, 10µL of the cellular fraction after centrifugation, was placed on a microscope slide in the middle of the marked 2 cm x 2 cm square area and covered with a coverslip. The samples were then viewed with a Zeiss Axio Observer 7 fluorescent microscope with the MetaCyte Metafer Classifier CoolCube1 camera and an EC Plan-Neofluar 40x/0.75 M27 objective (Carl Zeiss Microscopy, Munich, Germany). The excitation wavelength for PAC-1 was again set at 450 to 488 nm and the emission at 499 to 529 nm (demarcated as the green fluorescence), and for the CD62P-PE marker excitation was set at 540 nm to 570 nm and the emission at 577 nm to 607 nm (demarcated now as the orange fluorescence).

Four slides were loaded at a time and analysis was done using the automated MetaSystems Metafer software (Version 4.3, MetaSystems, Altlußheim, Germany). We developed a novel MetaCyte Metafer classifier for platelets on this software and our proposed area and threshold parameters are shown in Table 3. This minimum object area was chosen as it represents the smallest size that allows for effective platelet detection while minimizing background artifacts, ensuring accurate segmentation and classification. However, we acknowledge that this threshold might lead to the exclusion of some of the smallest individual platelet sizes. However, this conservative approach was followed to minimize background artifacts.

**Table 3:** Proposed area and threshold parameters using the automated metasystems metafer software (Version 4.3, metasystems, altlußheim, Germany)

**Single Cells area**	
Minimum Object area	16.00µm2
Maximum Object area	750.00µm2
Max. Concavity Depth	0.700
Maximum Aspect Ratio	3.500
**Thresholding**	
CS Object Threshold	5%
UT Sat Area (1/10µm2)	0%
Minimum Upper Thr.	0%
Dilate / Erode Cycles	2
**Second threshold**	
Minimum Threshold	8%
Threshold Increment	2%
Maximum Threshold	18%

Slides were captured in two channels with green fluorescence as counterstain for focusing and object detection and orange fluorescence for detailed analysis. In the first analysis step all objects below a certain counterstain threshold (green channel) are rejected as background artefacts. For remaining objects, a combination of integrated intensity and intensity distribution in the red channel within the objects contour was used for selection of true positives. Finally, objects were counted, and the total object area is summed up. Results were manually checked for any incorrectly identified particles and to highlight the platelets for reporting. A report was created in MetaSystems Neon Report Editor for the platelet results that includes the total area (µm²), mean roundness and total count of the platelets.

### Statistical analysis and data analysis

Analysis was done in Graphpad 10.3, R-statistical software (version 4.4.2) and Microsoft Excel. Data were arrayed in a suitable format for visual statistical analysis with the dplyr library in R and then further adjusted manually in Excel. Graphical representations were produced in R with the ggplot2 and ggsignif libraries.

## Results

See Table 2 for an overview of the demographic characteristics of the 25 participants from our study cohort. We documented the distribution of co-morbidities and self-reported symptoms. The Long COVID cohort in our study exhibited notable pre-existing comorbidities preceding their acute COVID-19 infection.

We deliberately chose not to normalize or dilute the samples, as our aim was to assess platelet morphology and activation in the samples as close to their native physiological state as possible, without introducing artifacts that could arise from manipulation or alteration of platelet concentrations. It is well recognized that platelet activation and clumping can be influenced not only by paracrine signalling, particularly in the presence of higher platelet densities, but also by circulating inflammatory molecules, including viral components such as spike protein [14]. These factors likely contribute to the significant platelet activation, spreading, and clumping observed in COVID-19 samples, and are important considerations in interpreting the differences in platelet size and area distributions between groups.

While the current study did not directly explore the potential correlation between hyperactivated platelet detection by correlating specific symptoms and comorbidities, this novel method may lay the groundwork for future studies to unravel the relationship between different platelet parameters and distinct symptoms.

Although not a major objective of this study, we added two platelet inhibitors, as addition confirmation that the preparation methods do not additionally activate platelets. After confirmation that the inhibitors did not prevent further platelet activation (See Supplementary Figs. 1 and 2), the rest of the samples were analysed using a manual micrograph capturing method, as well as a novel automated MetaCyte Metafer classifier method for platelets.

### Manual platelet visualization and automated metacyte metafer classifier for platelet analysis

**Fig. 1:**
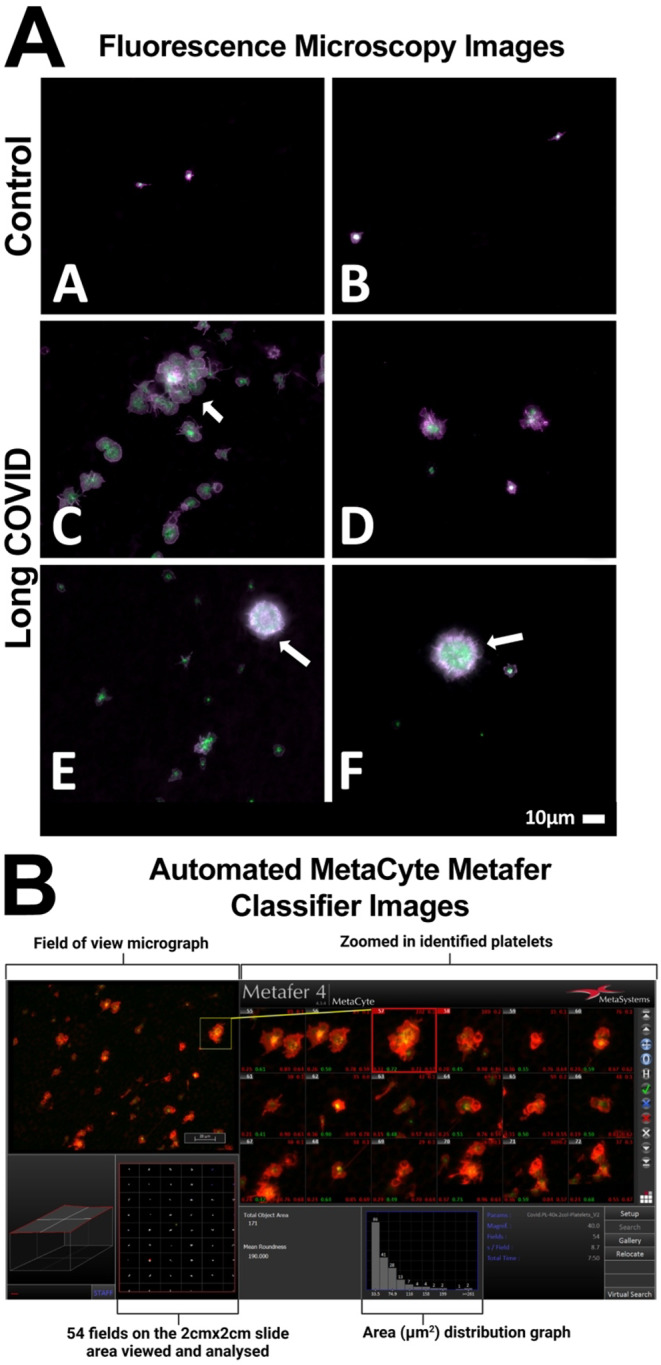
**A and B**: Platelets captured by fluorescence microscopy and the Automated MetaCyte Metafer Classifier. **1 A)**: Representative manually imaged fluorescence micrographs of control **(A and B)** (*n* = 10) and Long COVID **(C-F)** (*n* = 15) patients, where platelet activation can be seen with the platelet spreading and clumping (arrows) in the Long COVID samples compared to the controls. PAC-1: Green fluorescence; CD62P-PE: Purple fluorescence. **1B)**: Metasystem results screen, indicating the main field of view micrograph and the zoomed in identified platelets on the 2 cm x 2 cm area on the slied. The Metafer software also automatically calculates the area, roundness and count of each sample

**Fig. 2:**
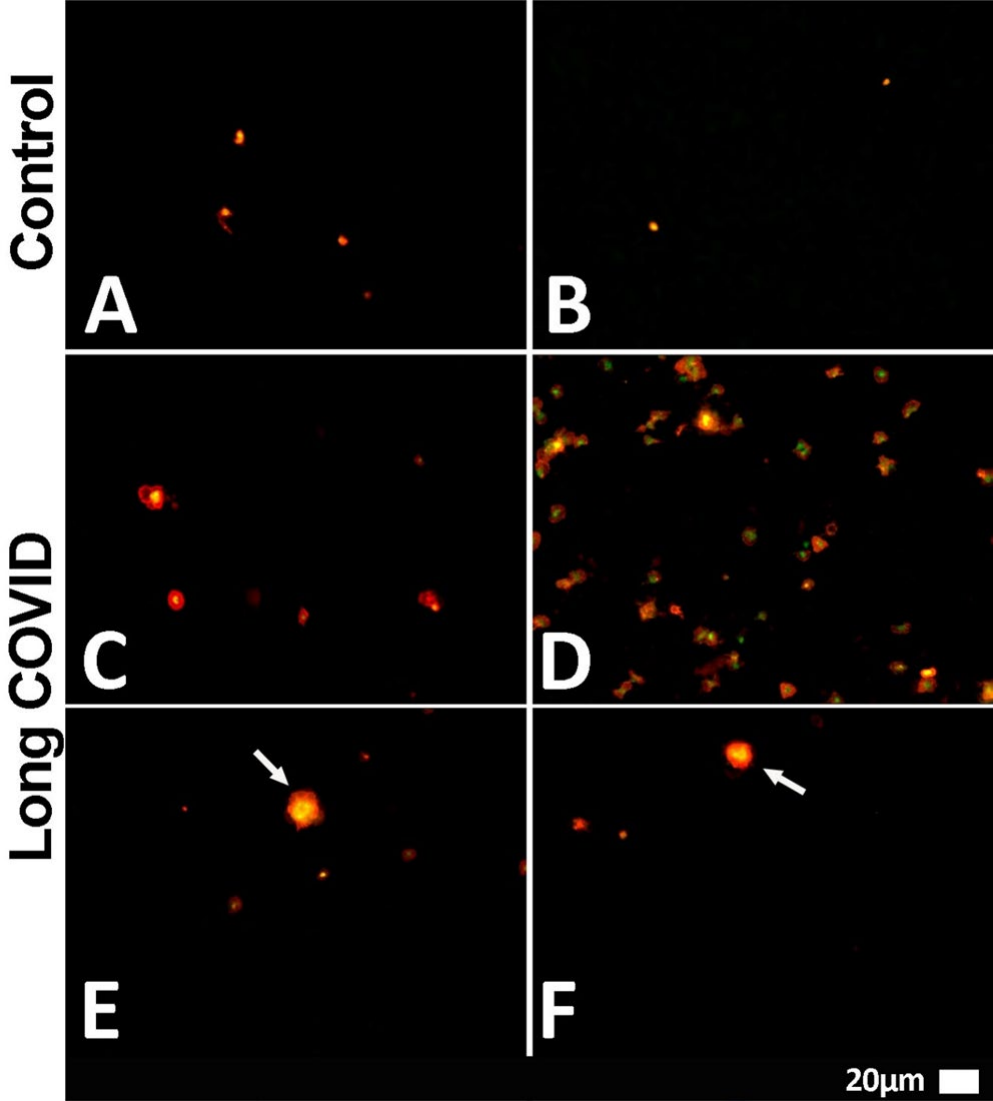
Representative fluorescence micrographs taken with the MetaCyte Metafer Classifier system of Control **(A and B)** (*n* = 10) and Long COVID **(C-F)** (*n* = 15) patients, where platelet activation can be seen with the platelet spreading and clumping (arrows) in the Long COVID samples when compared to the controls. PAC-1: Green fluorescence; CD62P-PE: Orange fluorescence

Figure 1A and B show platelets captured by fluorescence microscopy and the automated MetaCyte Metafer Classifier. Figures 1A (A and B) show the platelets form healthy participants. In healthy individuals, platelets are normally small and round with only a few pseudopodia extending from their surface. The platelets from the Long COVID patients showed moderate spreading [Figure 1A (C to F)] with some clumping present [Figure 1A (C, E, F) (arrows)]. Figure 1B is an example of a typical view of the MetaCyte Metafer Classifier window after the analysis of a microscope slide. Figure 2A to F show representative micrographs imaged and analysed by the MetaCyte Metafer Classifier automated system attached to the Zeiss microscope.

In the MetaCyte Metafer classifier analysis, we defined 14 discrete size ranges for platelet dimensions to accommodate varying conditions of platelet activation. These may include single platelets or platelet clumps, with size range 4–14 that can include both single platelets and platelet clumps. The initial three size ranges, categorized as sizes 1, 2, and 3, are indicative of the dimensions typically associated with platelets found in healthy controls samples. The subsequent ranges, from 4 to 13, were designed to represent the varied dimensions resulting from platelet hyperactivation and potential platelet clumping. The largest defined range, size 14, corresponds to the maximum platelet size, and potential platelet clumping, that might be encountered in a sample.

For analytical purposes, we further categorized these ranges into groupings based on expected platelet sizes. Typically, control platelets fall within size ranges 1 to 3. Consequently, for comparative analysis, we organized the size ranges into broader groups to illustrate typical versus atypical platelet size distributions.

These groupings include:

- Group 1–2 (16–57.14 μm²) representing typical platelets as seen in a healthy control sample;
- Group 1–3 (16–85.71 μm²) to include a slightly broader range of normal platelet sizes;
- and additional groups representing varying degrees of platelet activation (Groups 3–14, 4–14, etc.).

Refer to Table 4 for detailed delineations of these size range groupings and their corresponding platelet size intervals. We also performed a manual platelet grading using the micrographs from the automated Metasystems analysis and compared it to our manual grading system done using micrographs from our static fluorescence microscope. The spreading and clumping results for both techniques were found to be very similar. Manual analysis vs. automated analysis for spreading: healthy (1.9 vs. 2.15) and Long COVID (3.0 vs. 2.96). Manual analysis vs. automated analysis for clumping: healthy (1.0 vs. 1.1) and Long COVID (1.21 vs. 1.61) (See Supplementary Table 1).

**Table 4:** Platelet count size range grouping (1–14) and group selections

**Groups**		**Size ranges (µm** ^**2**^ **)**	
1		16–28.57	
2		28.57–57.14	
3		57.14–85.71	
4		85.71–114.29	
5		114.29–142.86	
6		142.86–171.43	
7		171.43–200	
8		200–228.57	
9		228.57–257.14	
10		257.14–285.71	
11		285.71–314.29	
12		314.29–342.86	
13		342.86–371.43	
14		≥ 371.43	
**Group selections**	**Size distribution (µm** ^**2**^ **)**	**Group selections**	**Size distribution (µm** ^**2**^ **)**
1–2	16–57.14	3–14	57.14 - ≥400
1–3	16–85-71	4–14	85.71 - ≥400
1–4	16–114-29	5–14	114.29 - ≥400
1–6	16–142.86	7–14	171.43 - ≥400

### Platelet distribution and size determination analysis using metacyte metafer classifier data

**Fig. 3:**
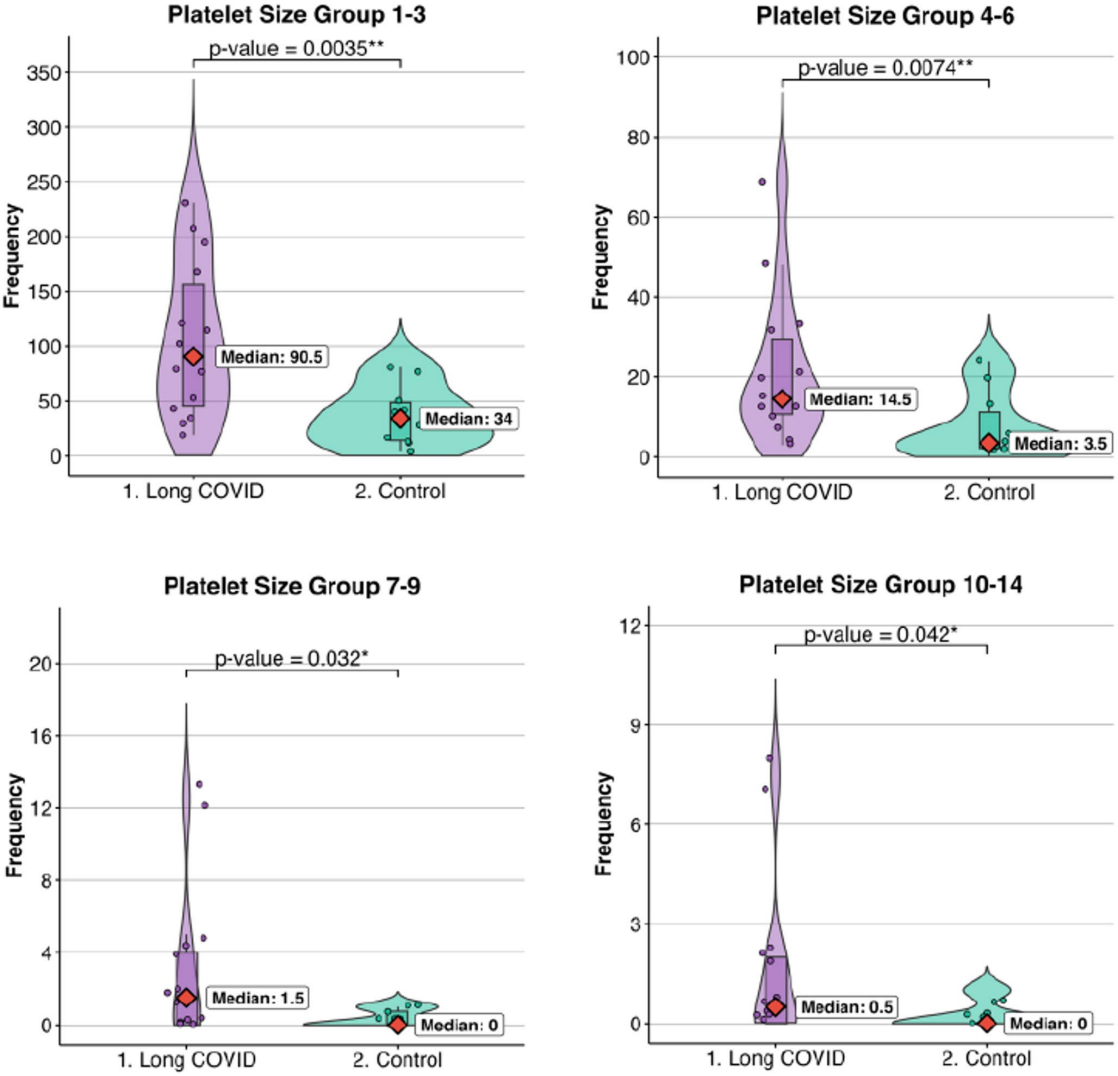
Violin-box plots of platelet size groups frequency between controls (*n* = 10) and individuals with Long COVID (*n* = 15). Medians and significance bars indicated in each panel. Jitter dots indicate individual samples by sample group. P-values were calculated using a Mann-Whitney U Test. Significance levels: *p* < 0.1 = *; *p* < 0.05 = **; *p* < 0.01 = ***. The key insight from here is that platelet frequencies (count) are consistently and significantly higher across all size groups in Long COVID samples

Figure 3 presents violin plots of platelet size group frequency across different size groups for controls and participants with Long COVID, with jittered points and overlaid boxplots providing additional granularity. Frequency is calculated as the count of platelets within each size group observed in each sample over each sample type (control and Long COVID). The key insight from Fig. 3 is that platelet frequencies (counts) are consistently and significantly higher across all size groups in Long COVID samples.

Each panel in Fig. 3 displays two distributions: one for participants with Long COVID (purple) and one for controls (green), allowing for a direct visual comparison. The violin plots reveal clear differences between the groups, particularly in the smallest platelet size range (1–3), where Long COVID participants exhibit both higher median frequency and greater variability. The spread of the distribution suggests a broader range of platelet frequency among Long COVID patients, while control samples cluster more tightly around lower values.

As platelet size increases, the overall frequency decreases for both groups, but the decline is less subtle in the Long COVID sample. Long COVID participants maintain higher frequency across all size categories, though the gap between the two groups narrows with increasing platelet size. By the 10–14 size range, the distributions have converged, with platelet counts in Long COVID participants approaching those of the control group, though still slightly elevated.

For inference, we assess whether platelet frequency in different size groups follow a normal distribution and conducted Shapiro-Wilk normality tests separately for Long COVID participants and control samples. The results indicate that normality holds only for the smallest platelet size group (1–3), where both Long COVID (*p* = 0.208) and control (*p* = 0.305) samples fail to reject the null hypothesis, suggesting that their distributions are not significantly different from normality. However, for all larger platelet size groups (4–6, 7–9, and 10–14), p-values fall below conventional significance thresholds (*p* < 0.05), rejecting the null hypothesis in both Long COVID and control groups. This suggests deviations from normality, particularly in the larger platelet size categories, where the distributions become increasingly skewed or non-normally shaped.

Given these findings, we conducted one-sided Mann-Whitney U tests for each platelet size group (also shown in Fig. 3), as this non-parametric test does not assume normality and is well suited for comparing distributions with potential deviations from normality. The results provide strong statistical evidence that platelet size group frequency is significantly elevated in Long COVID participants relative to controls across all size categories.

The hypothesis tested:

$$\:{H}_{0}:{Count}_{Long\:COVID}=\:{Count}_{Control}$$

$$\:{H}_{a}:\:{Count}_{Long\:COVID}>\:{Count}_{Control}$$

For platelet size groups 1–3, the test yielded a p-value of 0.0035, indicating a highly significant difference in distribution between the two groups. A similar trend was observed in platelet size groups 4–6 (*p* = 0.0074), suggesting a consistent and substantial elevation in platelet size group counts among Long COVID participants in the smaller size ranges. The effect, while still statistically significant, was somewhat attenuated in the larger size categories. Platelet size groups 7–9 exhibited a p-value of 0.0317, and platelet size groups 10–14 approached the conventional significance threshold with *p* = 0.0424, indicating that while platelet frequency remains elevated in Long COVID participants, the magnitude of the difference diminishes as platelet size increases.

Due to the presence of ties in the data, exact p-values could not be computed, and an asymptotic approximation was used. Despite this limitation, the consistently low p-values across all size groups indicate a robust and statistically significant shift in platelet distribution among Long COVID participants, with the most pronounced differences occurring in the smaller platelet size categories. These findings suggest a systematic alteration in platelet size distribution in Long COVID, which reflects underlying pathophysiological mechanisms warranting further investigation. Table 5 summarises the inference results discussed above, along with additional descriptive statistics.

**Table 5:** Platelet group size selections when comparing controls with participants with long COVID. P-values calculated from Mann-Whitney U test

	Control (*n* = 10)		Long COVID (*n* = 15)	
Platelet Size Group Selections	Median [Q1-Q3]	*p*-value	Median [Q1-Q3]	Difference in Median Count
1–3	34 [14-48.75]	0.0035	90.5 [45.5-156.25]	56.5
4–6	3.5 [2.25–11.25]	0.0074	14.5 [10.75–29.25]	11
7–9	0 [0-0.75]	0.0317	1.5 [0–4]	1.5
10–14	0 [0–0]	0.0424	0.5 [0–2]	0.5

**Fig. 4:**
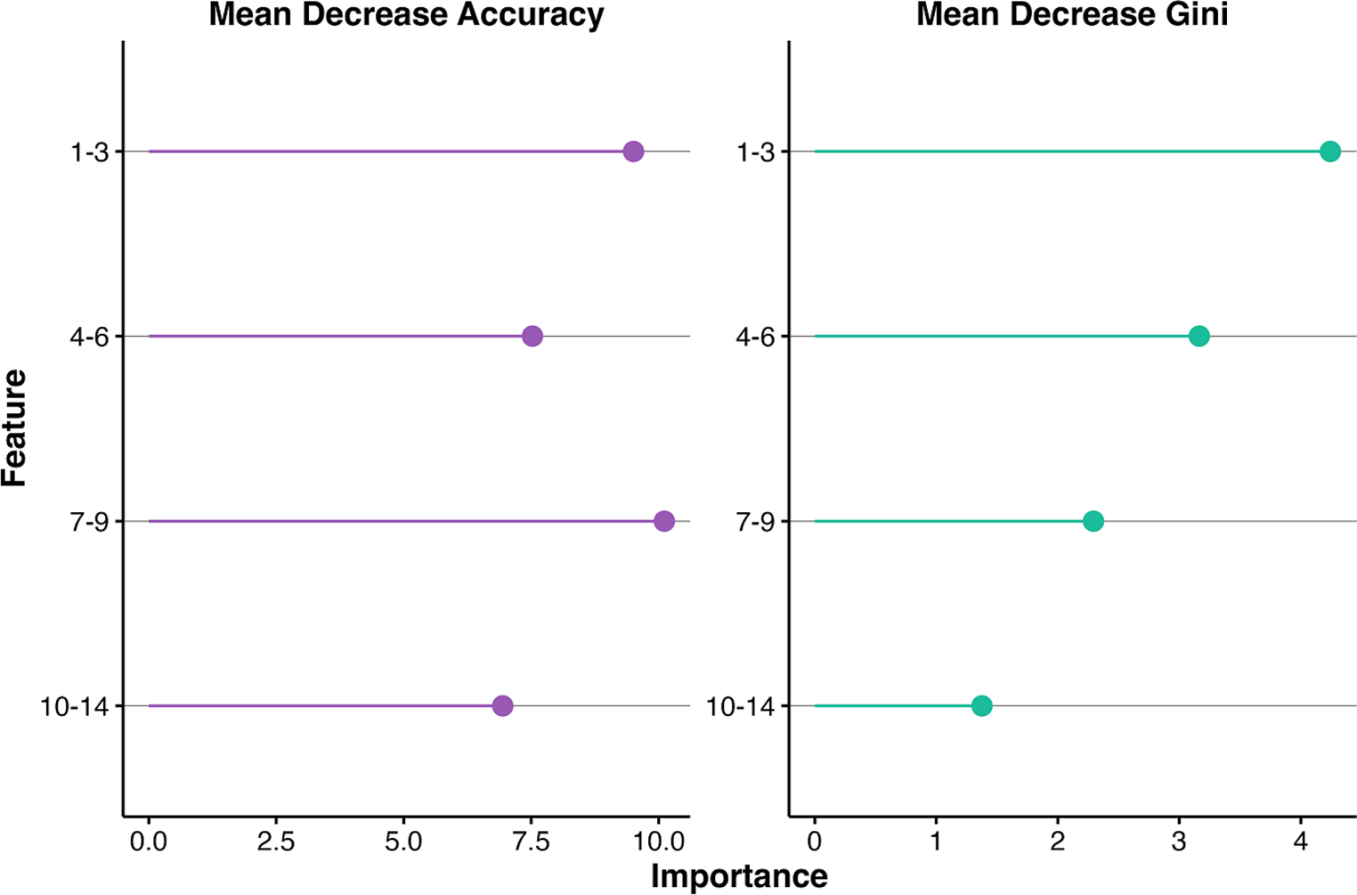
Size group importance in differentiating Long COVID (n = 15) from control samples (n = 10)

In our study, we further employed a simple random forest model to discern the most salient features for distinguishing between control subjects and Long COVID samples, with particular interest in identifying influential platelet size groups. The model’s variable importance was assessed using two metrics: Mean Decrease in Accuracy and Mean Decrease in Gini, shown in Fig. 4. This revealed that the 1–3 size group was paramount in both metrics, indicating its significant role in model accuracy and purity of node separation. Subsequent size groups (4–6 and 7–9) exhibited diminished importance, with the 10–14 size group contributing the least. This trend was consistent across both metrics, providing evidence that smaller platelet size groups are key predictors in differentiating between patient conditions. Notably, while both metrics converge on the relative ranking of feature importance, they differ in scale, reflecting their unique methodological underpinnings (mean Decrease in Accuracy pertains to predictive performance, whereas Mean Decrease in Gini relates to homogeneity within model nodes).

## Discussion

In our previous studies, we have shown that platelets are key role-players in coagulation pathologies and also in Long COVID [1, 2, 4, 5, 15–17]. Our fluorescence microscopy method has offered novel insights into platelet activation in inflammatory conditions, as evidenced by our development of a platelet grading system for hyperactivated platelets [7]. These findings align with the growing body of literature suggesting altered platelet dynamics in individuals with Long COVID. Platelet pathology were confirmed by a recent paper by Buonsenso and coworkers, using flow cytometry, where they found that, also in children with Long COVID, significant platelet pathologies underlie the condition [8].

In the current study, we wished to first confirm the robustness of our sample processing procedure. We first investigated whether the laboratory preparation method, using a centrifugation step, might cause platelet activation. We added PAC1 as our marker to identify platelets and CD62P-PE as a marker of platelet degranulation and activation. In future studies, we may consider including CD41 alongside CD62P-PE and PAC-1 to complement these activation-specific markers and provide a more comprehensive assessment of both platelet presence and activation states.

Here we showed that platelets are already activated in circulation of Long COVID patients, as the addition of platelet inhibitors (Indomethacin and PGE-1), did not have any significant effect on the ultrastructure of platelets. To further study the inhibitory effects of Indomethacin and PGE-1, a larger and more robust analysis should be done.

While fluorescence microscopy provides valuable insights into structure and morphology, questions often arise regarding the conditions under which the images were captured and analysed. Another important consideration is analyst bias, that is significantly influenced by the level of expertise of the analyst capturing microscopy images. High-throughput imaging and analysis methods have been developed by various companies, and is therefore now more accessible, making it possible for faster, more accurate imaging, with minimal human intervention. Such analysis methods might have potential for implementation in both research and clinical settings.

We thus compared manual microscopy image capturing, by an experienced analyst, with an automated morphology analysis using the MetaCyte Metafer classifier. Both methods consistently identified significant platelet activation in the Long COVID group. The automated MetaSystems classifier also demonstrated statistical significance across all platelet size groupings when comparing controls with Long COVID participants, underscoring its effectiveness. Moreover, the classifier’s ability to provide detailed and unbiased metrics, such as area, count, and roundness, in a high-throughput manner marks a significant advancement in microscopy platelet analysis. These metrics offer a novel perspective on platelet structure, potentially revealing subtle changes in platelet function that traditional manual micrograph capturing methods may overlook. There is often attenuation bias brought on by measurement error in the manual analysis. Therefore, we opt for automated analysis.

In conclusion, our findings indicate that the MetaCyte Metafer classifier delivers accurate, consistent, and unbiased results. The automated MetaCyte Metafer classifier system demonstrated significant utility in analyzing platelet activation across a wide range of size categories. By defining 14 discrete size ranges, the system enables a comprehensive assessment of platelet dimensions, capturing variations sizes seen during various stages of platelet activation. This capability allows for a detailed investigation of activation patterns, which might not be as evident using traditional manual methods. The precision in segmenting platelet size groups provides high-resolution insights into the progression of platelet activation, thereby highlighting subtle morphological changes that could be crucial in identifying and understanding thrombo-inflammatory conditions like Long COVID. Furthermore, the classifier’s capacity to provide unbiased, high-throughput analysis enhances the reliability and reproducibility of results, making it a valuable tool for both research and clinical diagnostics. This approach not only aids in distinguishing between typical and atypical platelet morphology, but also helps identify distinct activation profiles that could be linked to disease severity and outcomes. In conclusion, the MetaCyte Metafer classifier is intended to complement, not replace, traditional platelet analysis methods, offering a robust and efficient tool to enhance the accuracy and depth of platelet structure and activation assessments. Future analyses using Metasystem software could benefit from the development of additional image processing parameters, tailored to assess specific platelet morphological parameters, such as roundness and aspect ratios. Implementing such metrics may enhance the precision of morphological comparisons between sample types. In addition, a smaller minimum object area threshold can also be explored to assess whether a refined range allows for more precise platelet segmentation while maintaining the exclusion of background artifacts. Thus, future studies can build on these findings by refining the size range definitions and incorporating artificial intelligence (AI) to further optimize data interpretation and clinical relevance.

## Electronic supplementary material

Below is the link to the electronic supplementary material.

Supplementary Material 1

## Data Availability

No datasets were generated or analysed during the current study Please change this to: Datasets are available on request and in supplementary material.
