# Supplementary material for: A novel metacyte metafer classifier for platelet morphology using long COVID as a model
Source: J Thromb Thrombolysis. 2025 Jul 11;59(3):664–75. doi: 10.1007/s11239-025-03144-9 (PMC13246536; doi:10.1007/s11239-025-03144-9)

SUPPLEMENTARY FIGURES AND TABLES

Supplementary Table 1: Manual and automated MetaCyte Metafer classifier analysis of platelet spreading and clumping.

**Manual platelet grading analysis**		
	**Spreading**	**Clumping**
Control (n=10)	1,9	1,0
Long COVID (n=15)	3,0	1,21
**Automated MetaCyte Metafer classifier analysis**		
	**Spreading**	**Clumping**
Control (n=10)	2,15	1,1
Long COVID (n=15)	2,96	1,61

Supplementary Figure 1: Representative fluorescence micrographs taken by manual fluorescence microscopy of a Long COVID patient before (control) and after the addition of the platelet inhibitors [Indomethacin and Prostaglandin E1 (PGE-1)], where platelet activation can be seen unchanged before and after the addition of the platelet inhibitors. PAC-1: Green fluorescence; CD62P-PE: Purple fluorescence. Statistical analysis was performed on the percentage area covered by platelets, calculated as the proportion of total micrograph area (using ImageJ 1.53a). A one-way ANOVA was conducted (using Graphpad Prism 10) to compare the % area across the three experimental groups (Control, Indomethacin, and PGE-1). No statistically significant differences were observed ($p = 0.11$).

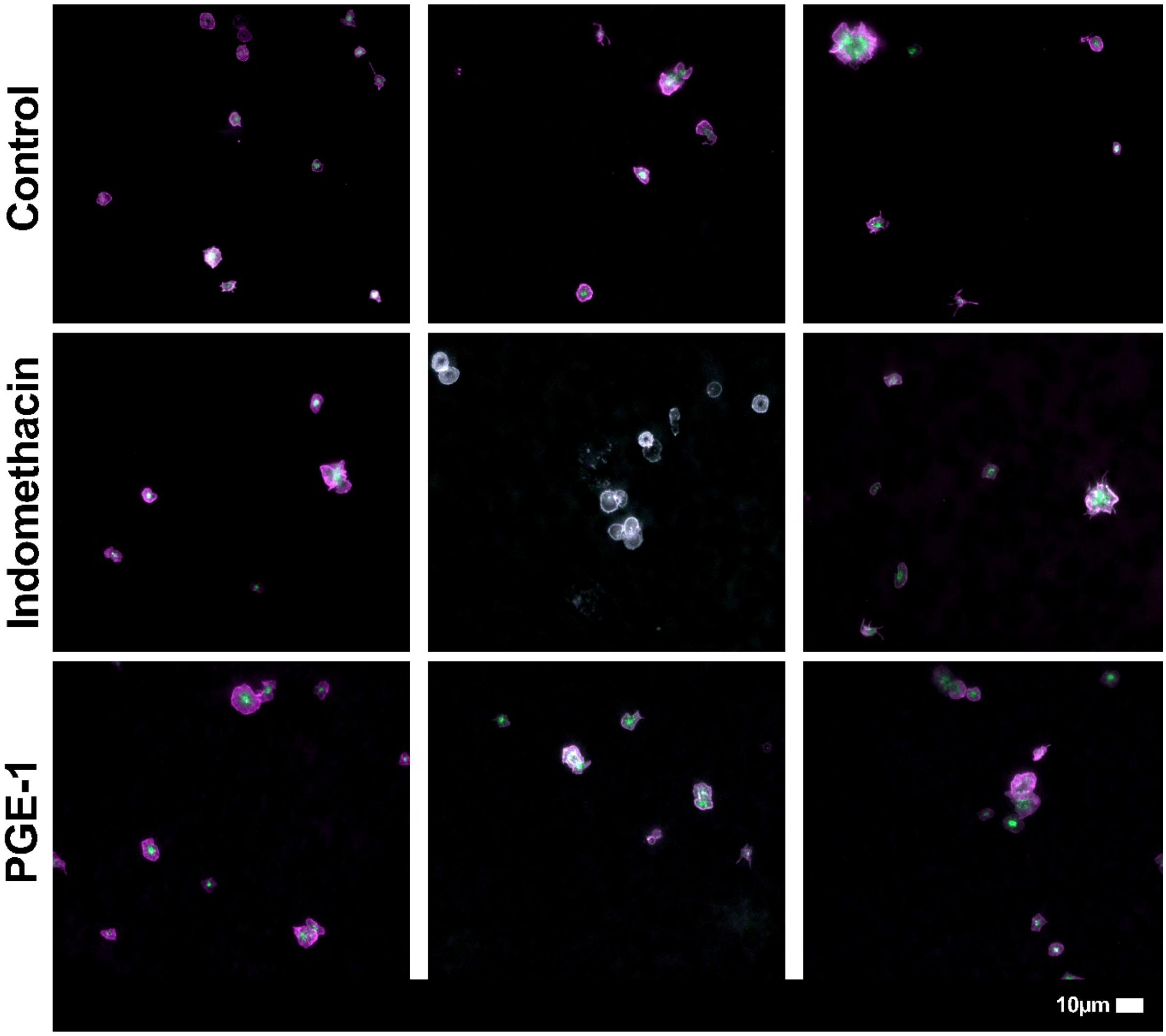

Supplementary Figure 2: Representative fluorescence micrographs taken with the MetaCyte Metafer Classifier system of a Long COVID patient, before (control) and after the addition of the platelet inhibitors [Indomethacin and Prostaglandin E1 (PGE-1)], where platelet activation can be seen unchanged before and after the addition of the platelet inhibitors. PAC-1: Green fluorescence; CD62P-PE: Orange fluorescence. Statistical analysis of the percentage area covered by platelets was performed using data obtained from the MetaCyte Metafer Classifier system (using ImageJ 1.53a). A one-way ANOVA was used to compare the control, Indomethacin, and PGE-1 groups (using Graphpad Prism 10). No significant differences were detected between the groups ($p = 0.06$).

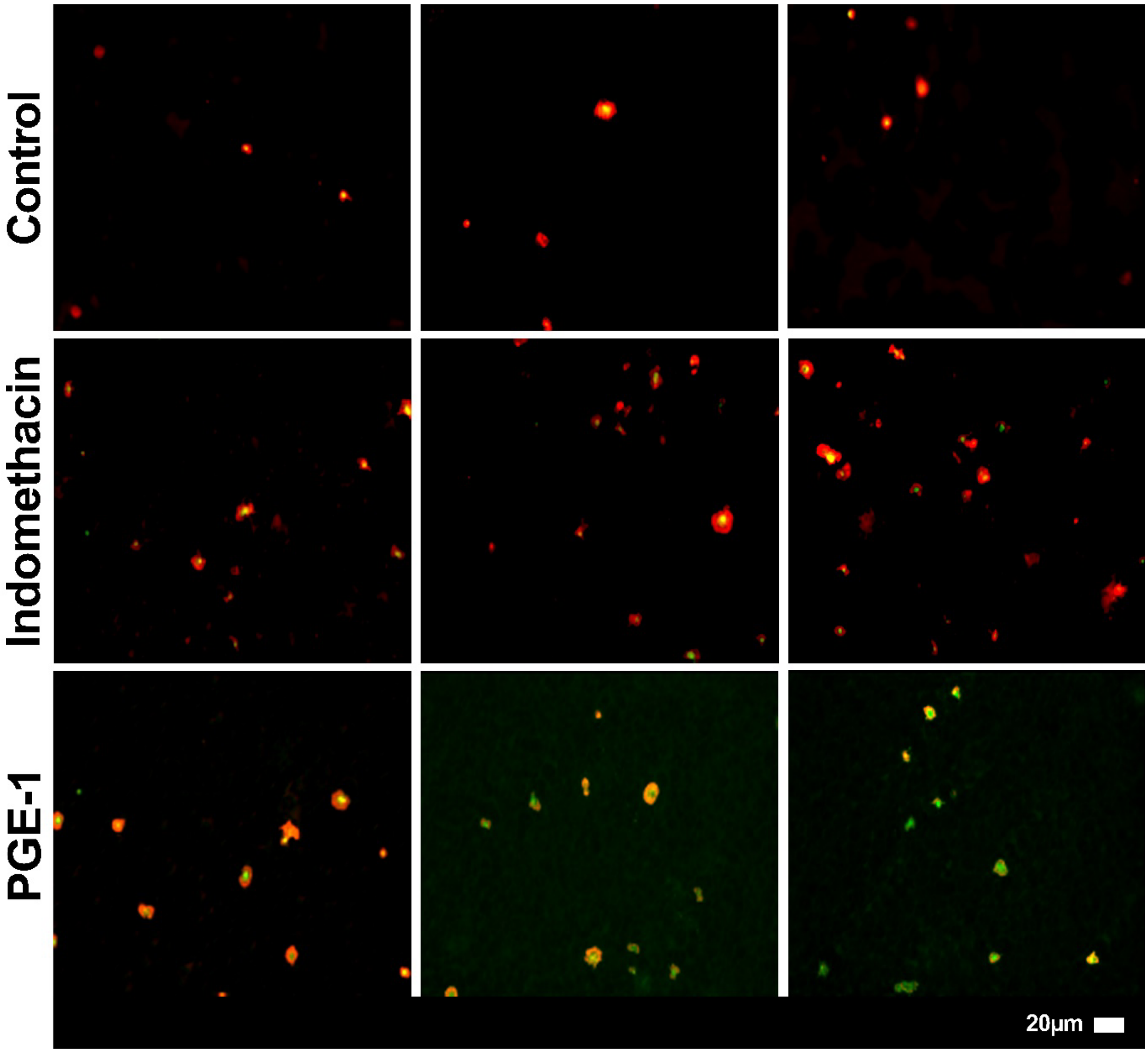

Supplement: Supplementary file 1 — Supplementary Material 1 [file 11239_2025_3144_MOESM1_ESM.pdf]
